# X-ray-induced hydride formation in palladium nanowires in a gaseous hydrogen environment

**DOI:** 10.1107/S1600576726004620

**Published:** 2026-06-15

**Authors:** Hanna Sjö, Huaiyu Chen, Giuseppe Abbondanza, Dmitry Dzhigaev, Megan O. Hill Landberg, Jesper Wallentin, Johan Gustafson

**Affiliations:** a Synchrotron Radiation Research, Department of Physics Lund University 221 00 Lund Sweden; b NanoLund, Department of Physics Lund University 221 00 Lund Sweden; c Department of Physics Chalmers University of Technology 412 96 Gothenburg Sweden; d MAX IV Laboratory Lund University 221 00 Lund Sweden; Goa University, India

**Keywords:** metal hydrides, hydrogen, beam damage, nanowires, powder diffraction

## Abstract

Palladium hydrides are promising systems for hydrogen sensors and hydrogen storage, making the study of hydride formation in nanoparticles highly relevant. Lately, such studies have often been performed *in situ* or *operando* using synchrotron radiation of increasingly high intensity and brilliance. While beam-induced effects are well documented in liquid environments, they are rarely considered in the gaseous phase. Here, X-ray diffraction is used to investigate hydride formation in palladium nanowires, comparing hydrogen-induced transitions with transitions triggered by varying X-ray exposure rates. Although hydrogen exposure naturally drives hydride formation, we observe a clear additional contribution from beam-induced effects. These findings highlight the need to account for such effects when studying gas–solid reactions with X-rays.

## Abstract 2

Powder diffraction is used to reveal X-ray-induced hydride formation in palladium nanowires in a gaseous hydrogen environment.

## 1. Introduction

Hydrogen has a wide range of possible applications, among others as an alternative fuel (Crabtree & Dresselhaus, 2008 ▸; Singla *et al.*, 2021 ▸; Ramachandran & Menon, 1998 ▸). A hydrogen-dependent future requires progress in safe and efficient storage, given the dangers associated with storing it as a liquid or gas (Usman, 2022 ▸), as well as reliable hydrogen sensing technologies (Wadell *et al.*, 2014 ▸). Metal–hydrogen (M–H) systems, often consisting of hydrides, are promising and thus well studied in different combinations and forms. Metal hydrides are particularly intriguing because they can achieve hydrogen densities even greater than liquid hydrogen, and without the same safety concerns as high-gas-pressure storage (Sakintuna *et al.*, 2007 ▸). One example is the palladium–hydrogen (Pd–H_*x*_) system, which offers many advantages in hydrogen storage and sensing, particularly in the form of nanostructures (Konda & Chen, 2016 ▸; Kumar *et al.*, 2019 ▸; Zhao *et al.*, 2015 ▸; Wadell *et al.*, 2014 ▸).

The Pd–H_*x*_ system undergoes distinct phase transformations from a dilute interstitial solid solution (α phase), at low hydrogen concentrations, to a lattice-expanded β phase, as the hydrogen concentration increases. Both phases retain a face-centred cubic structure, where hydrogen occupies empty octahedral sites. The α phase only results in a minor, nearly linear expansion with increasing hydrogen concentration, while the β phase is associated with an approximately 3% lattice expansion (Manchester *et al.*, 1994 ▸; Suzana *et al.*, 2021 ▸; Zhao *et al.*, 2015 ▸). These transformations are accompanied by lattice mismatches, inducing significant strain. The structure of the material plays a large role in the phase transition, where the surface and defects in the nanomaterial have a great impact (Pundt & Kirchheim, 2006 ▸; Goltsova, 2006 ▸). At intermediate hydrogen concentrations, the α and β phases have been reported to coexist (Suzana *et al.*, 2021 ▸).

Understanding the structural adaptations of crystalline lattices to accommodate solute atoms is crucial for these applications. Since the morphology of the material has been shown to influence hydride formation, and experimental techniques risk altering the material properties (Pundt & Kirchheim, 2006 ▸), careful consideration must be given to the fact that the measurement itself can induce hydride formation. The M–H formation in nanomaterials is often studied with X-ray diffraction (XRD) and other X-ray techniques (Holade *et al.*, 2015 ▸; Rose *et al.*, 2003 ▸; Johnson *et al.*, 2019 ▸; Bugaev *et al.*, 2017 ▸; Muduli *et al.*, 2024 ▸). Therefore, investigating the interplay between beam effects and hydride formation is essential, especially since synchrotron X-ray sources can produce focused beams with high intensity. In similar measurements in aqueous solutions, the energy is mainly absorbed in the water, leading to radiolysis, where the higher H presence at the surface can then affect the kinetics of the transition to the β phase and thus explain beam-induced hydride formation (Björling *et al.*, 2023 ▸; Maillard *et al.*, 2025 ▸; Atlan *et al.*, 2025 ▸). The use of high-flux measurements to study nanoparticles in aqueous solution without influence on the result has thus been deemed very difficult (Björling *et al.*, 2023 ▸; Atlan *et al.*, 2025 ▸). Given that even in an atmospheric pressure of gaseous H_2_ radiolysis is assumed to be minimal due to the minor energy absorption, beam-induced hydride formation has not been predicted in gaseous environments.

In this work, we investigate the effects of X-ray measurement on Pd–H*_x_* formation in a gaseous environment. The samples studied here are electrochemically grown metallic nanowires in porous alumina (PAA) (Larsson *et al.*, 2020 ▸). XRD is used to compare the α-to-β phase transition at different X-ray doses and H_2_ exposure times. A comparison of time-dependent and measurement-rate-dependent transformations reveals how the measurements affect this system. We find that the transition to the denser β phase is significantly faster in areas of high X-ray exposure, showing that it is necessary to consider beam-induced effects also in gaseous environments.

## 2. Experimental details

### 2.1. Sample and environment

**Figure 1:**
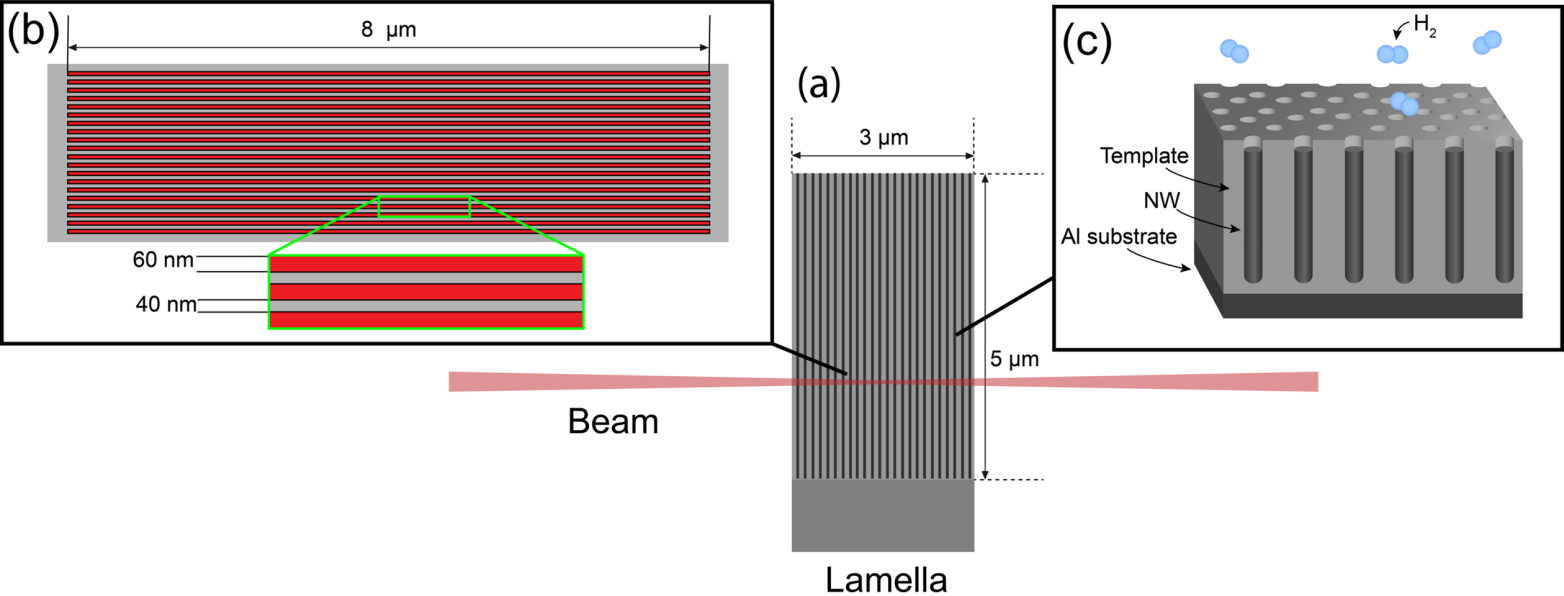
Sample and measurement procedure. (*a*) Illustration of the lamella and focused beam. (*b*) The measurement procedure of one area. One scan consists of 20 lines measured continuously. The 100 nm vertical steps give a line distance of 40 nm as the beam is 60 nm in this direction, as shown in the green rectangle. (*c*) Illustration of a section of the PAA sample (NW: nanowire).

The sample of polycrystalline Pd nanowires in a porous alumina template was grown as previously described by Larsson *et al.* (2020 ▸, 2021 ▸). Similar samples have previously been used in hydride studies (Abbondanza *et al.*, 2023 ▸). The nanowires had a diameter of 40 nm, interpore spacing of 65–100 nm and fixed lengths of approximately 5 µm. The alumina template was then milled to a lamella with approximately 3 µm thickness by focused ion beam milling at the Lund Nano Lab (Dzhigaev *et al.*, 2020 ▸). Sketches of the lamella geometry are shown in Figs. 1 ▸(*a*) and 1 ▸(*c*). The lamella was placed in a cell as described by Ulvestad *et al.* (2015 ▸).

The environment was controlled by a gas system based on Bronkhorst EL-FLOW mass flow controllers, with a maximum flow of 50 ml_n_ min^−1^, and a back-pressure controller, EL-PRESS set to a pressure of 100 mbar, connected to a vacuum pump with base pressure of 10^−2^ mbar. The environment switched between vacuum and a flow of H_2_ diluted to 2% in Ar, resulting in a H_2_ partial pressure of 2 mbar. The volume of the whole system was about 50 ml, such that it, at 100 mbar, takes about 0.1 min to reach the set pressure after starting the H_2_ exposure.

### 2.2. X-ray diffraction

The measurements were performed at the NanoMAX beamline at the MAX IV Laboratory (Johansson *et al.*, 2013 ▸). A photon energy, *E*, of 15 keV was used, and the beam was focused to 60 × 59 nm (vertical × horizontal). The photon flux, *f*, was 1.54 × 10^9^ photons s^−1^. The diffraction signal was measured using a Pilatus3 X 1M detector (981 × 1043 pixels, 172 µm pixel size and 1000 µm-thick Si sensor) at a sample–detector distance of approximately 158 mm. The sample–detector distance and direct-beam–detector position were calibrated using a NIST Si standard and *pyFAI-calib* (Kieffer & Karkoulis, 2013 ▸). The X-ray parameters are summarized in the supplementary information (SI) Table S1.

Five areas of the lamella were measured in a simultaneous series of measurements to compare the effects of the X-ray exposure with the naturally occurring hydride formation. All areas were measured before H_2_ exposure. Within 20 cycles of measurements during H_2_ exposure, two areas (A_1_ and A_2_) were measured every cycle, one area (B) was measured every fourth cycle, and two areas (C_1_ and C_2_) were only measured before the H_2_ exposure and in the last of the 20 cycles. The A and C areas were duplicated for control purposes and to be able to follow the transition back to the α phase with and without beam for both A and C areas. Each measurement involved continuous scans (fly scans) along 8 µm lines in the **x** direction, comprising 80 measurements with 0.1 s exposure each. This line scan was then repeated 20 times in 100 nm steps in the **y** direction, as shown in Fig. 1 ▸(*b*). The resulting measurement time was approximately 2.7 min; however, including the time spent moving between areas, the time between measurements was 3.49 min. The X-ray dose used in the analysis is approximated as
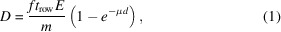
where *t*_row_ is the combined exposure time for a row in the measurement series, *m* is the approximate Pd mass within the measured row, μ is the attenuation coefficient for Pd at 15 keV and *d* is the average Pd thickness that the beam will pass through. In the dose approximation, the nanowires are assumed to be perfectly cylindrical and arranged in a hexagonal pattern. Only absorption in the nanowire is considered. Since the beamwidth is smaller than the step size in the **y** direction of the measurement series, the dose from the other rows is negligible, and the dose can be calculated for each measured row separately. The measured volume will thus be determined by the length of a measured line, the beam height (assumed to be 60 nm without further spread) and the thickness of the lamella. The resulting X-ray dose from one scan of an area is 63 MGy.

## 3. Results

**Figure 2:**
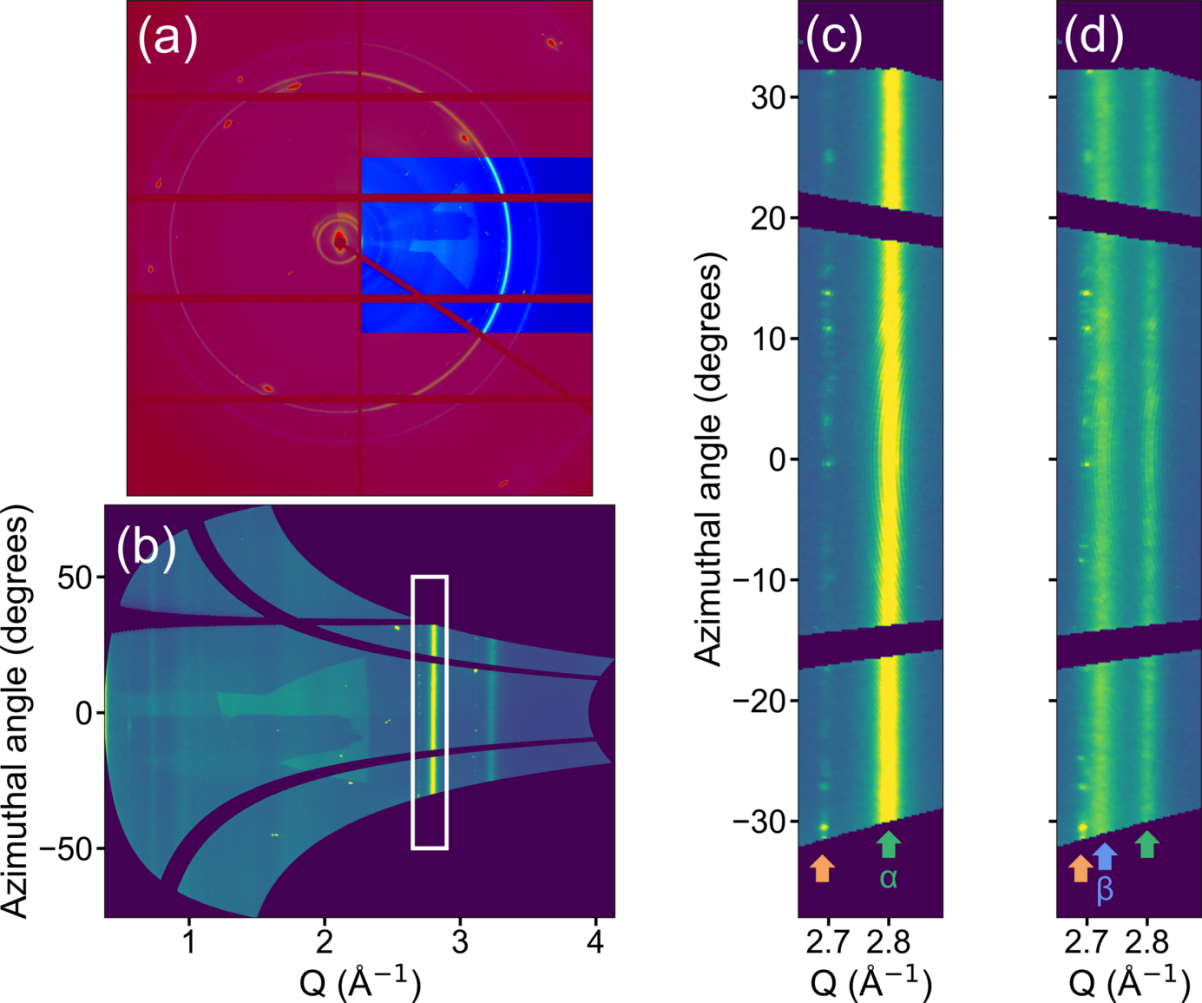
2D images of the diffraction. (*a*) Combined image showing the maximum intensity registered for each pixel for a measurement of area A_2_ before H_2_ exposure. The red area marks the mask used, and only the unmasked part is used in the later integration. (*b*) The unmasked area in (*a*) shown as reciprocal distance (*Q*) versus azimuthal angle. The area of interest is marked as the white rectangle. (*c*, *d*) The diffraction from area A_2_ within the white rectangle in (*b*) before H_2_ exposure (*c*), and after 152 min and 1200 MGy X-ray dose (*d*). The orange arrow marks a background line present in all measurements. The green and blue arrows mark the α line and β line, respectively.

Fig. 2 ▸(*a*) shows an example of a detector image collecting the diffraction pattern. To reduce background caused by strong Bragg spots from the Al substrate, the masked area is excluded from the analysis. Fig. 2 ▸(*b*) shows the used detector area as reciprocal distance (*Q*) versus azimuthal angle, which simplifies the powder rings to vertical lines. The lines of interest lie between 2.6 and 2.9 Å^−1^, within the area marked with the white box. Figs. 2 ▸(*c*) and 2 ▸(*d*) show the relevant part of the image before and after hydride formation for the A_2_ area. In Fig. 2 ▸(*c*), before H_2_ dosing, there is a single strong line at approximately 2.8 Å^−1^, corresponding to Pd(111). This line stays, with only a slight shift towards lower *Q*, during the α phase and is hence marked α. Fig. 2 ▸(*d*) shows an additional peak at approximately 2.73 Å^−1^, revealing the formation of the β phase. To validate the ring assignment, we assume that they correspond to the 111 reflections and calculate the lattice parameter *a* as
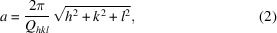
where *hkl* = 111. The α line corresponds to an average lattice parameter of 3.884 Å, consistent with the literature values for the Pd lattice constant. The β line is in general broader, but the centre corresponds to a lattice parameter of 3.989 Å. The average lattice expansion is 2.7%, which is at the lower end of what has previously been reported (Manchester *et al.*, 1994 ▸; Suzana *et al.*, 2021 ▸; Zhao *et al.*, 2015 ▸). In Figs. 2 ▸(*c*) and 2 ▸(*d*), there is an additional faint line at about 2.69 Å^−1^ that is due to background diffraction from the Al substrate and will be included as a constant background in the later fitting.

**Figure 3:**
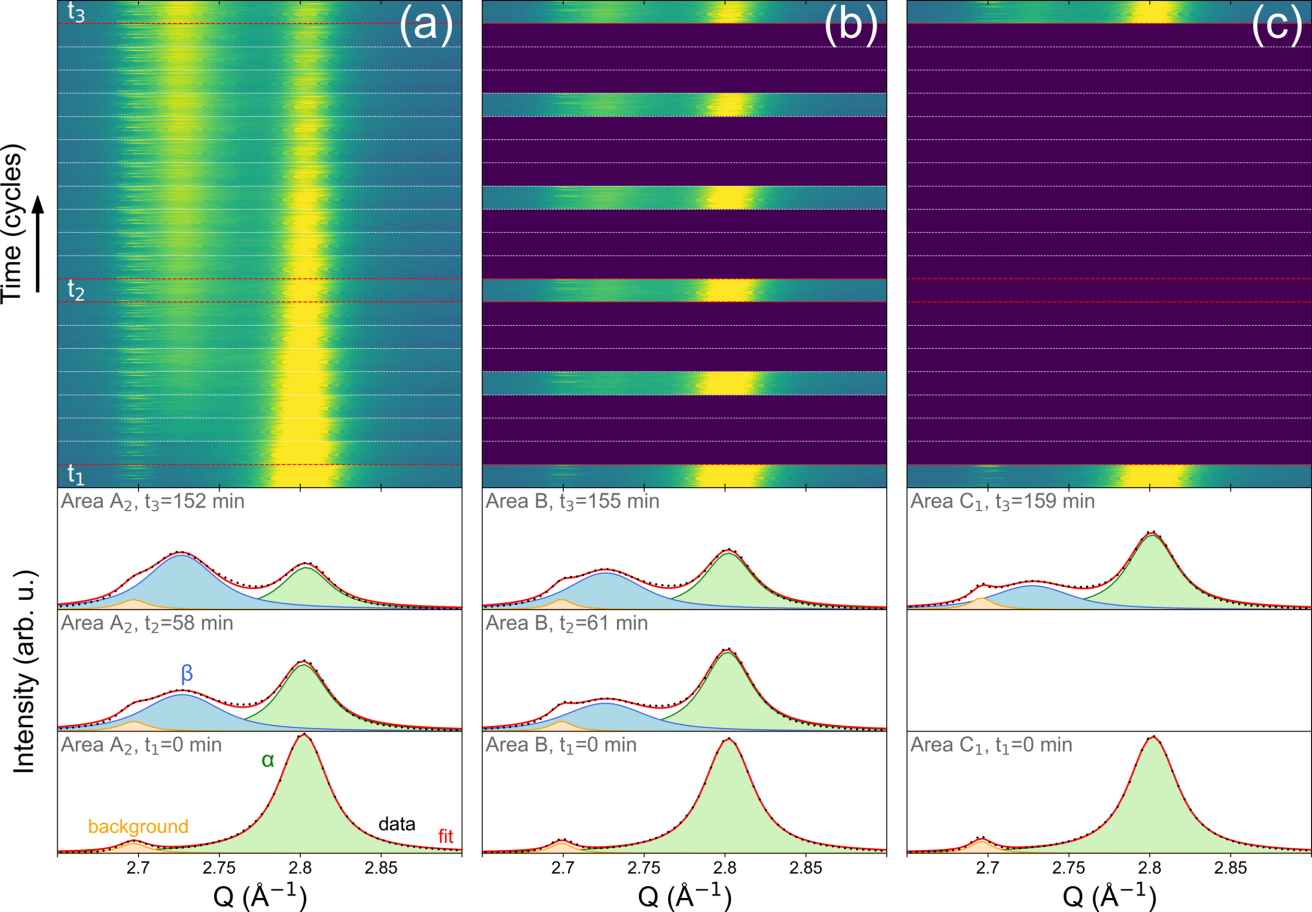
Peak fitting of integrated data. The top of each image shows the integrated intensity from an azimuthal angle range of −10–15°. Each slice corresponds to one measurement cycle. The timestamps marked with red are shown as a fit to the integrated intensity below. All measurements after *t*_1_ are during H_2_ exposure. The bottom of the figure shows the fitted integrated intensity with the data after subtraction of a linear background (black), total fit (red), α peak (green), β peak (blue) and substrate diffraction peak (orange). (*a*) Results from area A_2_, measured every cycle (high repetition rate). The bottom of the figure shows the fits of area A_2_ measured at the times *t*_1_ = 0, *t*_2_ = 58 min and *t*_3_ = 152 min. No β peak is visible before exposure, while it is clearly present after 58 min and larger than the α peak at the end of the measurement. (*b*) Results from area B, measured every fourth cycle (intermediate repetition rate). The fitted integrated intensity is from *t*_1_ = 0, *t*_2_ = 61 min and *t*_3_ = 155 min. (*c*) Results from area C_1_, measured just before and at the end of the H_2_ exposure (low repetition rate). The fitted integrated intensity is from *t*_1_ = 0 and *t*_3_ = 159 min. There is no *t*_2_ since the area was only measured twice. The β peak for area C_1_ is smaller than that for the other areas but still present, indicating a beam-induced effect on the phase transition but not a complete dependence.

Fig. 3 ▸ shows the X-ray diffraction data integrated along the azimuthal angle, as well as fits to follow the development of the α and β peaks. The shown area A_2_ was measured 20 times during H_2_ exposure, B was measured five times, and C_1_ was only measured before and at the end of the H_2_ exposure, hence purposefully introducing gaps in the data shown in Figs. 3 ▸(*b*) and 3 ▸(*c*). The lower part of Fig. 3 ▸(*a*) shows the fit of A_2_ at three different times (see SI Section 2 for fitting parameters). At *t* = 0, before the exposure to H_2_, there is no indication of a β phase. After 58 min of H_2_ exposure and 470 MGy X-ray dose, the two phases coexist, but the α peak is still dominant. Finally, after 152 min of H_2_ exposure and 1200 MGy X-ray dose, the β phase is dominant. In the lower part of Fig. 3 ▸(*b*), the corresponding time stamps for area B are shown, but now with a lower X-ray dose since the measurement rate is lower. At the intermediate time, the two phases coexist, just as for the A area, but the α phase is more dominant compared with the corresponding fit in Fig. 3 ▸(*a*). The β phase is never dominant for the B area during the timeframe of the measurement. For area C_1_ in Fig. 3 ▸(*c*), there is no intermediate measurement, but after the full H_2_ exposure, the β peak is present but weak.

**Figure 4:**
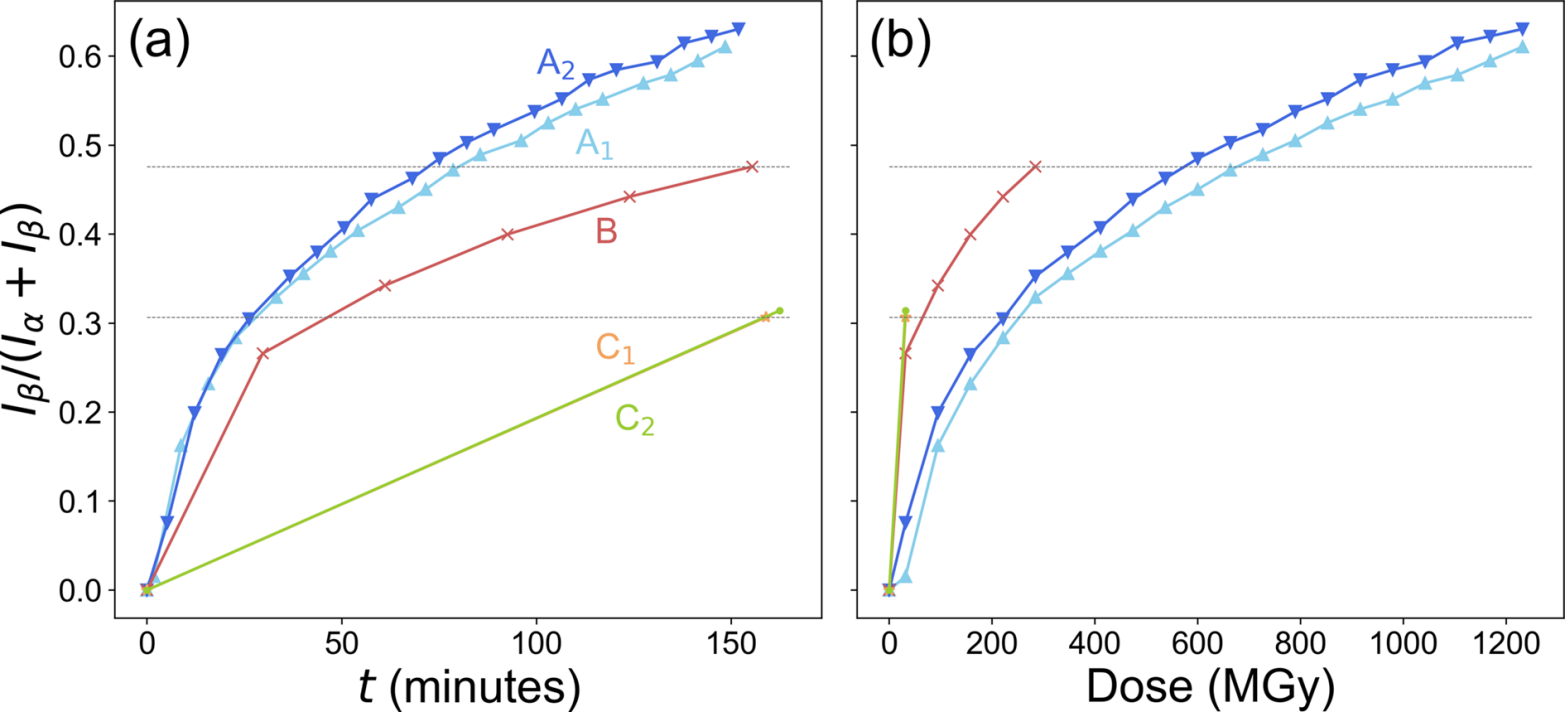
Integrated intensity depending on time and dose. (*a*) The fraction of integrated peak intensities of the β and α peaks as a function of time since the start of H_2_ exposure. *t* = 0 shows the measurement before exposure. The A areas, measured with a high repetition rate, show an accelerated phase transition and less plateau within the timeframe compared with area B, which is measured at a lower repetition rate. (*b*) The fraction of integrated peak intensities, now shown as a function of accumulated X-ray dose. Accumulated dose is calculated as the cumulative sum of the constant dose delivered per scan. If the phase transition were purely beam driven, the trajectories for the area types (A, B and C) would collapse when plotted versus dose. Instead, differences remain between the measurement protocols, indicating a contribution from H_2_-induced phase transition, independent of beam exposure.

To compare phase transition induced by the X-ray beam with that driven by H_2_ exposure, the relative area of the integrated β- and α-peak intensities (see Fig. 3 ▸ and SI Section 3 for fits) is shown for each region as a function of H_2_ exposure time and accumulated X-ray dose in Fig. 4 ▸. For the latter, the dose is calculated as the cumulative sum of the constant dose per scan (63 MGy). The dose associated with each point corresponds to the midpoint of that scan, calculated as the cumulative dose from previous scans plus half of the per-scan dose. The higher β fraction observed for areas A and B compared with C clearly shows that the measurements affect the hydride formation rate. Comparing the fractions for the last measurement for all areas can give a comparison at approximately the same H_2_ exposure time. The A areas have, at this point, been measured 15 and 19 times more in H_2_ compared with the B and C areas, respectively. At the final measurement point, the A areas show a β fraction of 130% relative to that of area B and 200% relative to that of the C areas. Furthermore, the β fraction reached at the final measurement of the B area is obtained in the A areas after approximately half the H_2_ exposure time. Similarly, the final fraction of the C areas was reached in the A areas in 1/5 of the time and in the B area in 2/5 of the time.

However, the phase transition is not purely measurement driven. This can be observed from the differences in trajectories for the areas when shown as a function of the accumulated dose. At the first measured point after the exposure to H_2_, the areas have received the same X-ray dose but experienced different H_2_ exposures. The B and C areas are measured after longer H_2_ exposure compared with A_2_ and show β fractions of 350% and 410%, respectively, relative to that of area A_2_. Area A_1_, on the other hand, has at the time of the first measurement received a lower H_2_ exposure and, correspondingly, has a β fraction that is 20% of that of A_2_.

**Figure 5:**
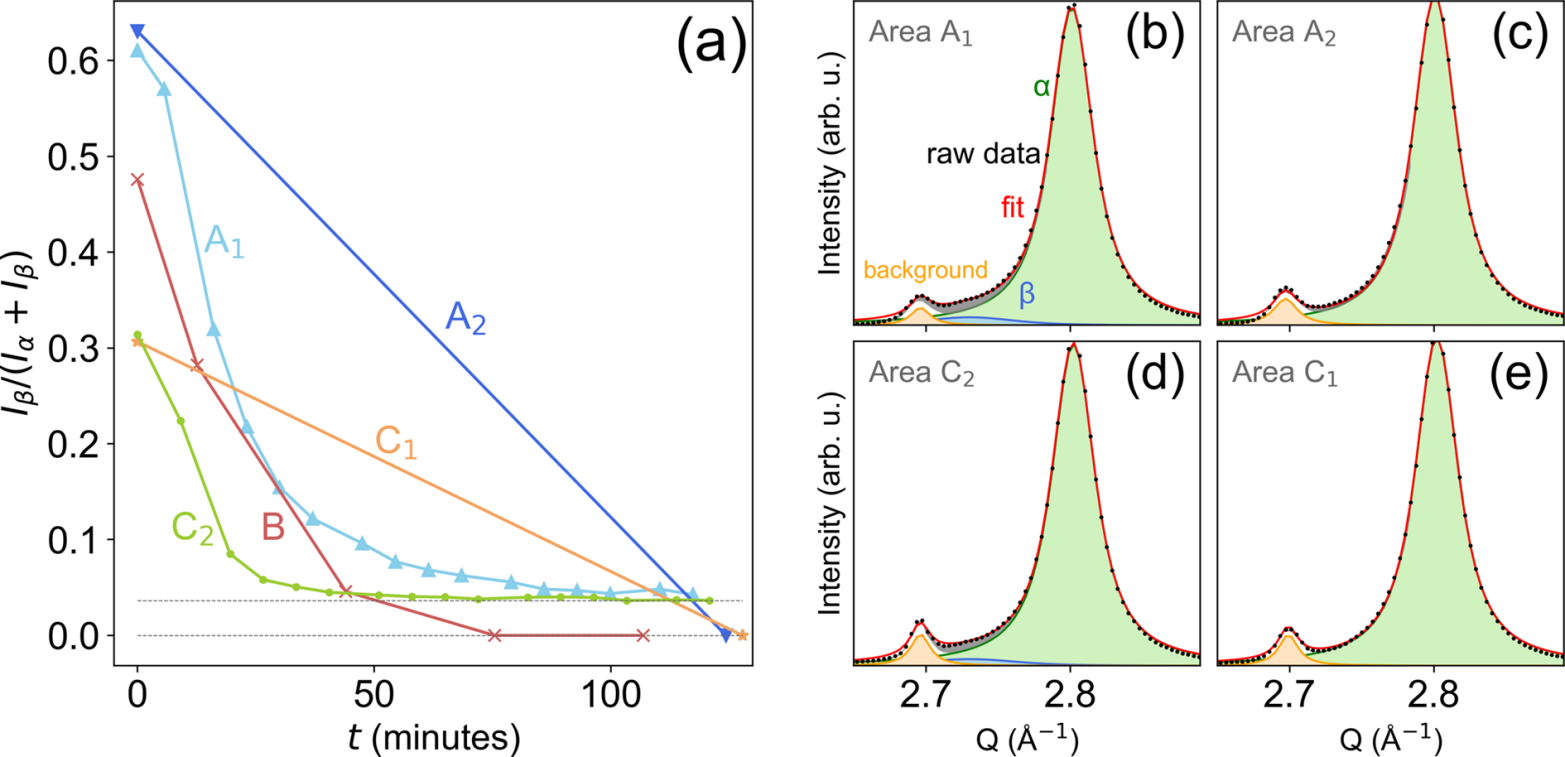
Reversed effects after the end of H_2_ exposure. (*a*) The fraction of integrated peak intensities of the β and α peaks for the five measured areas plotted against time since the end of H_2_ exposure. *t* = 0 shows the last measurement before the end of H_2_ exposure. (*b*–*e*) Fitted integrated intensity for the last measurement of four areas. Area A_1_ (*b*) and C_2_ (*d*) are measured with a high repetition rate, and A_2_ (*c*) and C_1_ (*e*) are only measured once at the end. The grey area marks the difference between this measurement and the fit of the measurement before H_2_ exposure (*t*_1_ in Fig. 3 ▸), which emphasizes the shoulder in (*b*) and (*d*), indicating that there is a non-zero β peak.

To examine the reversed effects, a comparable measurement procedure was initiated following the removal of H_2_ from the chamber. Here, the areas A_1_ and C_2_ were measured in all cycles, area B was still measured every fourth cycle, and areas A_2_ and C_1_ were only measured after all cycles. The β fractions of the different areas are shown as a function of time after the end of H_2_ exposure in Fig. 5 ▸(*a*). As expected, there is an onset in the β-to-α transition for all areas after the end of the H_2_ exposure. While the β phase of the samples with low X-ray exposures (A_2_, B and C_1_) disappears completely, there is still a shoulder in the integrated data of the constantly irradiated areas (A_1_ and C_2_), which could indicate a non-zero β fraction. The shoulder is visualized in the integrated data for the A and C areas, shown in Figs. 5 ▸(*b*)–5 ▸(*e*). Although the β component is small, there is a clear difference between the fitted measurements in Figs. 5 ▸(*b*) and 5 ▸(*d*) compared with Figs. 5 ▸(*c*) and 5 ▸(*e*). It is also striking that the β fraction stabilizes at nearly the same level of about 4% in both constantly measured areas. However, the small size of this shoulder introduces uncertainty in the peak position. Although it strongly resembles the smaller β peaks observed during the initial state of α-to-β transition, it cannot be completely excluded that the shoulder originates from another crystalline structure.

The steady state is reached after five cycles for area C_2_, which initially had a lower β fraction, and after nine cycles for area A_1_. Since the initial fraction varies between the areas, we do not find any obvious differences in the speed of the β-to-α transition. Depending on the amount of initial β phase, the β fraction becomes stable after about 50–70 min. So, there is a fairly rapid decrease of β phase after the end of the H_2_ exposure, but a high measurement rate sustains a small fraction for almost two hours. Since there seems to be a plateau, this small peak appears to be permanent, at least as long as the areas are measured at this rate. No measurement was performed after a longer time delay, so it is unknown if the state is permanent even without X-ray exposure.

## 4. Discussion

We have studied the beam dependence of the formation and stability of Pd–H_*x*_ by following the α-to-β and β-to-α transitions using XRD. By comparing the α and β components in parts of the sample measured at different rates, spontaneous and beam-induced hydride formation can be distinguished. It is evident that the hydride forms both with and without the X-ray beam, but the beam increases the transition rate significantly. For the β-to-α transition, there is no clear impact of the beam on the transition rate, but in all areas measured at a higher rate, the structure does not fully return to the initial state. Several possible mechanisms could explain the observed effects. However, the limited time resolution of our experiment prevents any detailed kinetic analysis. Even in the regions of higher acquisition frequency, our temporal resolution remains of the order of minutes, which is insufficient to capture the fast nucleation and growth dynamics expected for hydrides in Pd. In regions measured more sparsely, the lack of intermediate data points further complicates interpretation.

In the formation of M–H systems, different aspects can be rate determining depending on the system and experimental conditions (Bloch & Mintz, 1997 ▸). Heating caused by beam absorption could enhance hydrogen mobility. However, the expected hydrogen diffusion time in Pd under our experimental conditions is well below the temporal resolution of the measurements (Abbondanza *et al.*, 2023 ▸), suggesting that bulk diffusion is not rate limiting. Furthermore, elevated temperature is known to reduce both hydrogen uptake and hydride stability (Wadell *et al.*, 2014 ▸). Consequently, although a temperature increase during hydrogen uptake cannot be excluded, it is unlikely to account for the observed reaction behaviour.

This suggests that the limiting factor is unlikely to be bulk hydrogen transport but rather the nucleation process or the availability of hydrogen at the Pd surface. The X-ray beam may lower the energetic barrier for hydride nucleation, for instance, if beam-induced crystalline defects (beam damage) act as preferential sites (Bloch & Mintz, 1997 ▸; Pundt & Kirchheim, 2006 ▸) for hydrogen uptake. Such damage could accelerate the transition once sufficient hydrogen is available in the system. This could also explain the non-zero β component for the areas measured frequently during the β-to-α transition. We do not observe any effect in the area that was measured frequently during the α-to-β transition but sparsely during the β-to-α transition, indicating that previous irradiation does not impact the system. If beam damage underlies the beam-induced effects, the lack of a long-term impact might be due to defect healing as previously described in nanoparticles (Ulvestad & Yau, 2017 ▸), or that the beam damage is just a partial factor in the beam-induced hydride formation.

In addition to beam damage, radiolysis of adsorbed H_2_ could also contribute. Localized cracking of hydrogen mol­ecules under X-ray irradiation could increase the supply of atomic hydrogen at the surface, thereby promoting hydride formation. Furthermore, the emission of photoelectrons from the Pd sample or heating from the X-ray absorption could alter the local environment, such as excited interband states, or assist in the dissociation of adsorbed hydrogen, further influencing the kinetics of hydride formation.

The suggested effects are not mutually exclusive and may act simultaneously, with their relative importance depending on the rate-limiting step under the present conditions. If nucleation is the bottleneck, beam-induced damage and defect generation may dominate. If hydrogen availability is limiting, then beam-driven dissociation and electron-mediated effects could play a larger role.

The results of the reversed transition, without H_2_ in the chamber, are challenging to analyse due to the varying and relatively low temporal resolution, as well as the different initial states of the measured areas. The remaining intensity at the position of the β peak, in the high-dose cases, suggests that a small fraction of retained hydride persists in the crystal structure. Such behaviour could arise from local variations in defect density or irradiation-induced modifications of the kinetics that slow the β-to-α transition, allowing a small fraction of the β phase to remain stable under conditions where it would otherwise disappear. However, alternative explanations cannot be excluded. For instance, irradiation-induced lattice strain or local lattice expansion may lead to a slight peak asymmetry that shifts part of the intensity toward the β-phase position, producing a weak shoulder without the presence of a β phase. Alternatively, the additional peak could stem from another structure with similar lattice expansion that could also be promoted by the X-ray exposure, such as PdC_*x*_ from impurities in the chamber or sample. Because the observed signal is weak and we lack information on the amount of H_2_ in the crystal structure, it is not possible to unambiguously distinguish between these scenarios.

To place our findings in context, we compare our estimated X-ray doses with those reported in similar studies where the hydrogen environments are comparable to the conditions used in our study. We have estimated the doses in these studies on the basis of the available information using the same principle as in the estimation of our X-ray dose. In a study by Suzana *et al.* (2021 ▸), nanoparticles of approximately 200–300 nm in diameter were measured with Bragg coherent diffraction imaging (BCDI). They report facet-dependent hydride formation, with nucleation occurring preferentially at particle tips and along certain low-coordination surfaces. The estimated dose per measurement is about three orders of magnitude higher than in our nanowire measurements, considering sample geometry, beam parameters and exposure time. At such high fluxes, beam-induced processes such as defect generation cannot be excluded. However, their work does not address beam-induced processes explicitly, so this interpretation remains speculative. Also, the geometry of their nanoparticles differs markedly from that of our nanowires. Particle size, and thus surface-to-bulk ratio, directly impacts both the kinetics and thermodynamics of hydride formation (Atlan *et al.*, 2025 ▸).

In a study by Ulvestad & Yau (2017 ▸), Pd nanoparticles of 200–400 nm were investigated with BCDI under hydrogen gas. They observed that dislocations nucleated during the α-to-β transition and that these defects subsequently healed during the reverse process. This ‘self-healing’ behaviour highlights the dynamic role of defects in nanoscale hydride formation and suggests that repeated cycling can erase beam-induced structural damage. While their results emphasize intrinsic defect dynamics, the high doses associated with BCDI measurements could also have influenced defect generation or annealing. Since experimental beam parameters are not reported in sufficient detail, we cannot directly compare the dose with our study. While their nanoparticles show defect healing, such behaviour may be less relevant in nanowires due to their extended geometry and different surface-to-bulk ratio, which could alter both defect stability and transformation pathways.

Looking instead at a bulk Pd sample, in the X-ray diffraction study by Kawasaki *et al.* (2015 ▸), the dose per measurement will be about two orders of magnitude smaller compared with our measurements. Here, they did see some unexpected fluctuation of the β- and α-peak intensities, which they attributed to beam-position instability. The lower X-ray dose is consistent with the interpretation that the bulk system is less sensitive; however, subtle beam effects cannot be fully excluded.

Since the underlying mechanism of the beam-induced effects cannot be unambiguously determined in the present study, the transferability to other gas-phase *operando*X-ray experiments cannot be fully assessed. However, several factors are likely to influence the magnitude of such beam-induced effects. In particular, nanostructured systems with a high surface-to-volume ratio may be more sensitive, as surface-mediated processes as well as thermal and kinetic effects can become more pronounced. In addition, materials that are susceptible to defect formation or lattice strain under irradiation may exhibit stronger beam-induced modifications of their transformation kinetics. Finally, the well-defined α-to-β transition in the Pd–H system makes beam-induced effects comparatively easy to detect, whereas similar effects may be more difficult to observe in systems without such pronounced structural signatures.

## 5. Conclusion

Synchrotron-based X-ray diffraction measurements of Pd nanowires in a gaseous H_2_ environment were used to study the transition between α and β phases in Pd hydride formation. We observe a clear X-ray-induced contribution to hydride formation, in addition to the naturally occurring transformation driven by hydrogen exposure. The transition appears accelerated within the measurement timeframe. Since the α-to-β transition does not fully plateau, it cannot be determined whether the final equilibrium value is altered by the X-ray exposure or if only the transition rate is affected. In the reversed transition, there is no evident rate effect noticeable with our time resolution. Notably, the system does not fully return to the initial state when exposed to a high measurement rate. This could indicate that the beam stabilizes a small amount of β phase within the nanowires or that there is beam damage that explains the change in the diffraction pattern.

While the underlying mechanism behind this beam-induced effect is not fully resolved here, several factors could contribute, including excited interband states, enhanced defect formation or X-ray-stimulated surface reactions. The observed beam-induced acceleration of hydride formation underscores the need to consider X-ray dose effects even in gaseous environments, where such phenomena are often overlooked. The estimated X-ray dose in our measurement is not unusually high in the context of studying hydride formation in Pd nanoparticles. Although the studies discussed report no evident beam-induced effects, there is no compelling reason to assume that our system is unique in this respect. The development of advanced X-ray sources has enabled remarkable opportunities. Our results do not imply that such sources should be avoided, but they emphasize the importance of carefully considering measurement-induced effects on the very processes they aim to probe.

## Supplementary Material

Supporting information file. DOI: 10.1107/S1600576726004620/hat5019sup1.pdf

## Data Availability

Data will be made available on request.

## References

[bb1] Abbondanza, G., Grespi, A., Larsson, A., Dzhigaev, D., Glatthaar, L., Weber, T., Blankenburg, M., Hegedüs, Z., Lienert, U., Over, H., Harlow, G. S. & Lundgren, E. (2023). *Nanotechnology* **34**, 505605. 10.1088/1361-6528/acf66e 37666238

[bb2] Atlan, C., Chatelier, C., Ngoipala, A., Olson, K., Viola, A., Bellec, E., Grimes, M., Gilles, B., Qamar, M., Mrovec, M., Leake, S. J., Eymery, J., Schülli, T. U., Vandichel, M., Richard, M. I. & Maillard, F. (2025). *J. Am. Chem. Soc.* **147**, 25417–25428. 10.1021/jacs.5c05102 40637705

[bb3] Björling, A., Marçal, L. A. B., Arán-Ais, R. M. & Solla-Gullón, J. (2023). *J. Phys. Chem. C* **127**, 13877–13885.

[bb4] Bloch, J. & Mintz, M. H. (1997). *J. Alloys Compd.* **253–254**, 529–541.

[bb5] Bugaev, A. L., Guda, A. A., Lomachenko, K. A., Shapovalov, V. V., Lazzarini, A., Vitillo, J. G., Bugaev, L. A., Groppo, E., Pellegrini, R., Soldatov, A. V., van Bokhoven, J. A. & Lamberti, C. (2017). *J. Phys. Chem. C* **121**, 18202–18213.

[bb6] Crabtree, G. W. & Dresselhaus, M. S. (2008). *MRS Bull.* **33**, 421–428.

[bb7] Dzhigaev, D., Svensson, J., Krishnaraja, A., Zhu, Z., Ren, Z., Liu, Y., Kalbfleisch, S., Björling, A., Lenrick, F., Balogh, Z. I., Hammarberg, S., Wallentin, J., Timm, R., Wernersson, L. E. & Mikkelsen, A. (2020). *Nanoscale* **12**, 14487–14493. 10.1039/d0nr02260h 32530025

[bb8] Goltsova, M. V. (2006). *Int. J. Hydrogen Energy* **31**, 223–229.

[bb9] Holade, Y., Napporn, T. W., Morais, C., Servat, K. & Kokoh, K. B. (2015). *ChemElectroChem* **2**, 592–599.

[bb10] Kieffer, J. & Karkoulis, D. (2013). *J. Phys. Conf. Ser.* **425**, 202012.

[bb11] Johansson, U., Vogt, U. & Mikkelsen, A. (2013). *Proc. SPIE* **8851**, 88510L.

[bb12] Johnson, N. J. J., Lam, B., MacLeod, B. P., Sherbo, R. S., Moreno-Gonzalez, M., Fork, D. K. & Berlinguette, C. P. (2019). *Nat. Mater.* **18**, 454–458. 10.1038/s41563-019-0308-5 30858567

[bb13] Kawasaki, A., Itoh, S., Shima, K., Kato, K., Ohashi, H., Ishikawa, T. & Yamazaki, T. (2015). *Phys. Chem. Chem. Phys.* **17**, 24783–24790. 10.1039/c5cp02725j 26343885

[bb14] Konda, S. K. & Chen, A. (2016). *Mater. Today* **19**, 100–108.

[bb15] Kumar, A., Mohammadi, M. M. & Swihart, M. T. (2019). *Nanoscale* **11**, 19058–19085. 10.1039/c9nr05835d 31433427

[bb16] Larsson, A., Abbondanza, G., Linpé, W., Carlà, F., Mousley, P., Hetherington, C., Lundgren, E. & Harlow, G. S. (2020). *J. Electrochem. Soc.* **167**, 122514.

[bb17] Larsson, A., Abbondanza, G., Rämisch, L., Linpé, W., Novikov, D. V., Lundgren, E. & Harlow, G. S. (2021). *J. Phys. D Appl. Phys.* **54**, 235301.

[bb18] Maillard, F., Atlan, C., Chatelier, C., Ngoipala, A., Olson, K., Viola, A., Bellec, E., Grimes, M., Gilles, B., Leake, S., eymery, J., Schulli, T., Vandichel, M., Richard, M.-I., Qamar, M. & Mrovec, M. (2025). Preprint available at *Research Square*, https://doi.org/10.21203/RS.3.RS-5629485/V1.

[bb19] Manchester, F. D., San-Martin, A. & Pitre, J. M. (1994). *J. Phase Equilib.* **15**, 62–83.

[bb20] Muduli, R. C., Chen, Z., Guo, F., Jain, A., Miyaoka, H., Ichikawa, T. & Kale, P. (2024). *Energy Adv.* **3**, 2212–2219.

[bb21] Pundt, A. & Kirchheim, R. (2006). *Annu. Rev. Mater. Res.* **36**, 555–608.

[bb22] Ramachandran, R. & Menon, R. K. (1998). *Int. J. Hydrogen Energy* **23**, 593–598.

[bb23] Rose, A., Maniguet, S., Mathew, R. J., Slater, C., Yao, J. & Russell, A. E. (2003). *Phys. Chem. Chem. Phys.* **5**, 3220–3225.

[bb24] Sakintuna, B., Lamaridarkrim, F. & Hirscher, M. (2007). *Int. J. Hydrogen Energy* **32**, 1121–1140.

[bb25] Singla, M. K., Nijhawan, P. & Oberoi, A. S. (2021). *Environ. Sci. Pollut. Res.* **28**, 15607–15626. 10.1007/s11356-020-12231-8 33538968

[bb26] Suzana, A. F., Wu, L., Assefa, T. A., Williams, B. P., Harder, R., Cha, W., Kuo, C. H., Tsung, C. K. & Robinson, I. K. (2021). *Commun. Chem.* **4**, 64. 10.1038/s42004-021-00500-7 PMC9814609 36697569

[bb27] Ulvestad, A., Welland, M. J., Collins, S. S. E., Harder, R., Maxey, E., Wingert, J., Singer, A., Hy, S., Mulvaney, P., Zapol, P. & Shpyrko, O. G. (2015). *Nat. Commun.* **6**, 10092. 10.1038/ncomms10092 PMC4682038 26655832

[bb28] Ulvestad, A. & Yau, A. (2017). *Nat. Commun.* **8**, 1376. 10.1038/s41467-017-01548-7 PMC5680230 29123126

[bb29] Usman, M. R. (2022). *Renewable Sustainable Energy Rev.* **167**, 112743.

[bb30] Wadell, C., Syrenova, S. & Langhammer, C. (2014). *ACS Nano* **8**, 11925–11940. 10.1021/nn505804f 25427244

[bb31] Zhao, Z., Huang, X., Li, M., Wang, G., Lee, C., Zhu, E., Duan, X. & Huang, Y. (2015). *J. Am. Chem. Soc.* **137**, 15672–15675. 10.1021/jacs.5b11543 26636882

