# Supplementary material for: X-ray-induced hydride formation in palladium nanowires in a gaseous hydrogen environment
Source: J Appl Crystallogr. 2026 Jun 15;59(Pt 4):1005–12. doi: 10.1107/S1600576726004620 (PMC13440063; doi:10.1107/S1600576726004620)
Supplement: Supplementary file 1 [file j-59-01005-sup1.pdf]

JOURNAL OF
APPLIED
CRYSTALLOGRAPHY

Volume 59 (2026)

Supporting information for article:

X-ray-induced hydride formation in palladium nanowires in a gaseous hydrogen environment

Hanna Sjö, Huaiyu Chen, Giuseppe Abbondanza, Dmitry Dzhigaev, Megan O. Hill Landberg, Jesper Wallentin and Johan Gustafson

Supplementary information

1. X-ray parameters

Table S1: Experimental parameters for X-ray measurement. The instrumental drift is very small and its effect on the results is negligible.

X-ray beam energy	Flux	Beam size	Exposure time	Scan time	Sample detector distance	Calibrant
15 keV	1.54×10^9 photons/s	60×59 nm	0.1 s	2.7 minutes	158 mm	NIST Si

2. Fitting parameters

The peaks were fitted using a symmetric Voigt profile. A linear background was removed before fitting. The fit of the background peak was optimised for the first measurement (before H_2 exposure) and then kept constant for each area since it later overlaps with the β-peak and seems stable. The input variables for the two peaks of interest are shown in Tab. S2. Comparison of residuals were used to determine the existence of a β-peak. Ranges were set to allow for variation. No special treatment of strain was applied other than allowing for peak broadening. The result of the fits is shown in Tab. S3. Since the α- and β-peaks are well separated and the background is approximately linear over the fitted range, the resulting uncertainty in the relative peak area is small. In this case, the ratio is only weakly sensitive to background choice, since any small baseline offset affects both peak areas similarly. The same is true for the choice of fit model. Analysing the error propagation based on the uncertainties of the fitted peak area results in an uncertainty of 0.008 or less, which is insignificant relative to the clear distinction of the relative peak area between measurement types.

Table S2: Peak fitting parameters. The peak center and σ are listed with their applied constraints, while γ is listed as the applied fixed value.

Identified peak	Peak center	σ	γ
α-peak	2.78-2.82	0.005-0.015	0.016
β-peak	2.70-2.73	0.005-0.023	0.020

Table S3: Peak fitting result and calculated lattice parameter from peak center

Identified peak	Peak center/Q ($Å^{-1}$)	Lattice parameter (Å)
α-peak	2.8016±0.0008	3.8844±0.0012
β-peak	2.7279±0.0016	3.9894±0.0023

3. Fits for α-to-β transition

Table S4: Summarised area description and some dose and β-fraction metrics.

	A_1	A_2	B	C_1	C_2
**Rate**	Every cycle	Every cycle	Every 4th cycle	Only at the end	Only at the end
**Number of scans**	21	21	6	2	2
**Accumulated dose (MGy)**	1231	1231	284	32	32
**Time to β-fraction ~0.3 (min)**	33	26	63	158	162
**Final β-fraction**	0.61	0.63	0.48	0.31	0.31

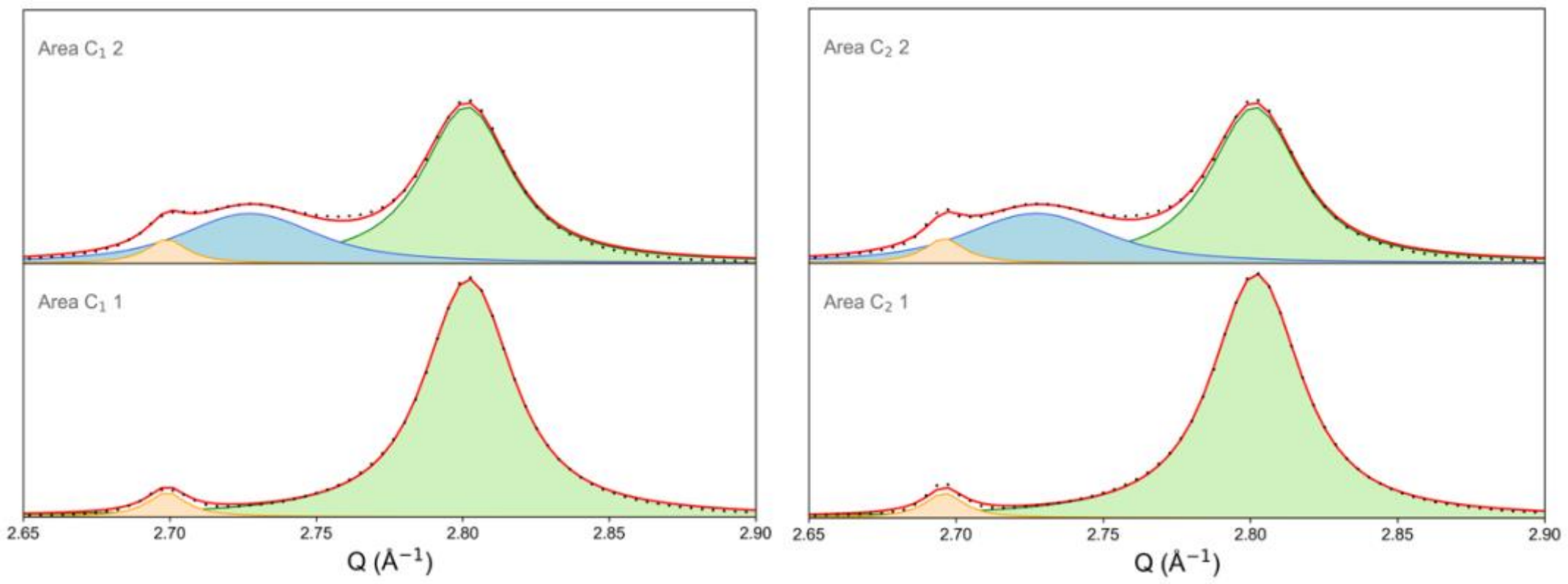

Figure S1: The fitted integrated data for area C_1 and C_2 with data after subtraction of a linear background (black), total fit (red), α-peak (green), β-peak (blue), and the substrate diffraction peak (orange).

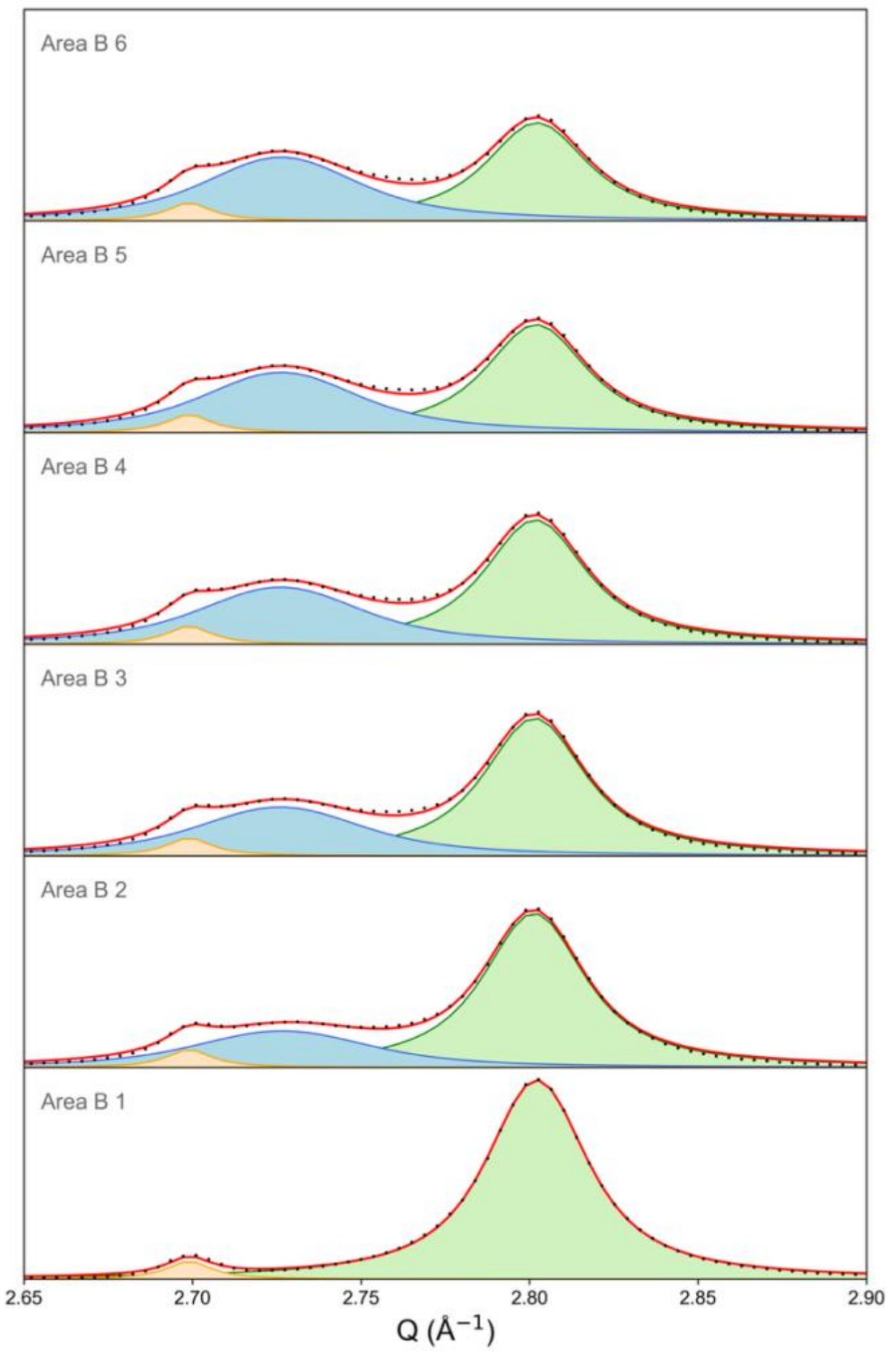

Figure S2: The fitted integrated data for area B with data after subtraction of a linear background (black), total fit (red), α-peak (green), β-peak (blue), and the substrate diffraction peak (orange).

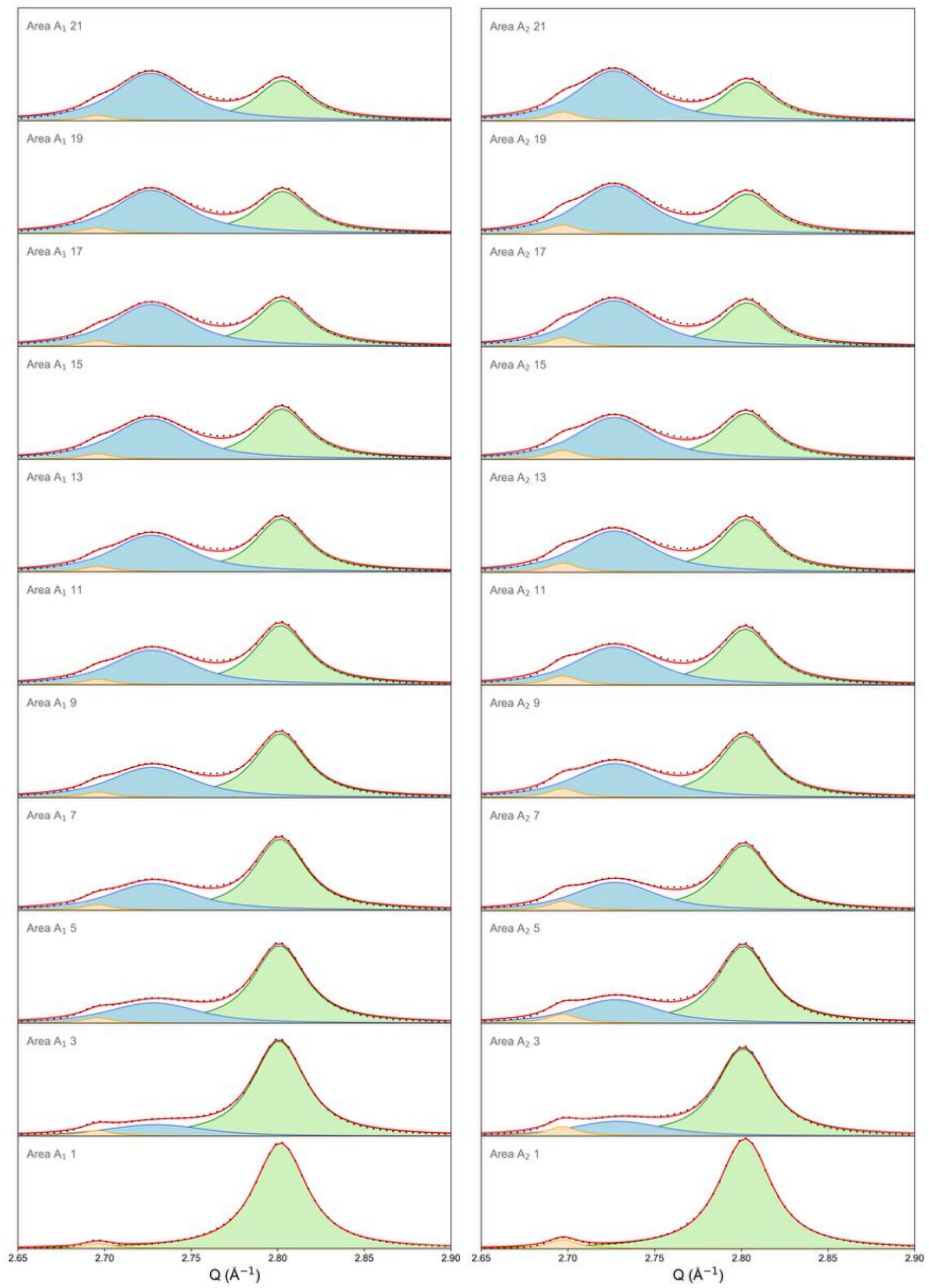

Figure S3: The fitted integrated data for area A_1 and A_2 with data after subtraction of a linear background (black), total fit (red), α-peak (green), β-peak (blue), and the substrate diffraction peak (orange). Every other measurement is shown.
